# Smaller babies at risk: birth weight impacts neonatal survival status in Silte zone, Central Ethiopia. A survival analysis of prospective cohort study

**DOI:** 10.3389/fped.2024.1426901

**Published:** 2024-12-12

**Authors:** Musa Jemal, Abdurezak Kemal, Bekri Mohammed, Delwana Bedru, Shemsu Kedir

**Affiliations:** ^1^ Department of Public Health, College of Medicine and Health Science, Werabe University, Werabe, Ethiopia; ^2^ Department of Public Health, College of Medicine and Health Sciences, Wolkite University, Wolkite, Ethiopia; ^3^ Department of Human Nutrition, Institute of Public Health, College of Medicine and Health Sciences, University of Gondar, Gondar, Ethiopia

**Keywords:** birth weight, neonatal mortality, survival status, Silt’e zone, Central Ethiopia

## Abstract

**Introduction:**

Globally, 2.4 million neonates died in their first month of life in 2019 with approximately 6,700 neonatal deaths every day. Ethiopia is 4th among the top 10 countries with the highest number of neonatal deaths. Yet, there are few prospective studies on neonatal mortality in the central region of Ethiopia. Hence, to develop evidence-based, locally tailored intervention strategies, it is necessary to evaluate neonatal survival status and mortality predictors, including birth weight. Therefore, the current study aims to assess survival status and factors predicting the survival of neonates in the Silt’e zone, Ethiopia.

**Methods:**

An institution-based prospective cohort study design was employed from 1 May to 30 July 2022. Data were collected from term neonates who were enrolled according to their order of health facility visit and then followed by data collectors in their homes. Data were analyzed using STATA version 14.1. Neonatal survival was presented using the Kaplan–Meier survival curve. The crude and adjusted associations were evaluated using the Cox proportional-hazards model, presented with a 95% confidence interval (CI), and a *P*-value <0.05 was used to declare statistical significance.

**Result:**

In total, 1,080 term neonates were followed for a total of 27,643.6 neonatal days. The study showed a 95% cumulative probability of surviving the neonatal period. The incidence rate of neonatal death was 2.02 per 1,000 neonatal days. Maternal history of neonatal death [adjusted hazard ratio (AHR) = 4.03; 95% CI: 2.28–9.52], complication during pregnancy (AHR = 3.08; 95% CI: 1.12–8.25), female sex (AHR = 0.45; 95% CI: 0.25–0.84), birth weight (AHR = 0.27; 95% CI: 0.11–0.63), and a low or intermediate APGAR score at 1 min (AHR = 3.11; 95% CI: 1.23–7.82 and AHR = 5.34; 95% CI: 1.63–17.51, respectively) were independent predictors of neonatal death.

**Conclusion:**

It has been noted that neonatal mortality in this area is higher than results from national studies and other study areas and thus requires strict attention and interventions targeting both the pre and postnatal periods. Babies with low birth weight were found to struggle to survive the neonatal period. Promoting maternal nutrition for normal birth weight of the newborn would thereby improve neonatal survival, and should be followed as a strategy.

## Introduction

The neonatal period, which starts at birth and ends at 28 completed days of life, is recognized as the riskiest time in newborns. The World Health Organization (WHO) defines neonatal mortality (NM) as “deaths among live births during the first 28 completed days of life,” which can be further classified as early neonatal deaths, between 0 and 7 completed days of life, and late neonatal deaths that occur between days 7 and 28 (1, 2).

The complex relationship between maternal, newborn, and healthcare-related conditions plays a role in increasing the likelihood of neonatal mortality. A high proportion of stillbirth and maternal and neonatal mortality occurs because of poor care during pregnancy and delivery (3). Hence, the neonatal mortality rate (NMR) is often used as a standard index for the evaluation of health, education, social systems, nutritional status, and health programs for neonates (4). A number of studies including a WHO report affirmed that the causes of most neonatal deaths were preterm birth (28%), intrapartum-related complications such as birth asphyxia (24%), sepsis (30%–50%), and birth defects (5–7).

Under-five mortalities decreased significantly from 5.0 to 2.4 million between 1990 and 2019. However, neonatal mortality rates decreased slower than post-neonatal child mortality. Currently, close to 1 million newborns die within the first 24 h, and the first week of life is a period in which three-quarters of all neonatal deaths occur (7).

Sub-Saharan Africa, with 27 deaths per 1,000 live births, had the highest NMR in 2019, followed by Central and Southern Asia with 24 deaths per 1,000 live births (7). Preceded by India, Nigeria, and Pakistan in decreasing rank, Ethiopia is 4th among the top 10 countries with the highest number of neonatal deaths with a total of 99,000 newborn deaths in 2019 (8). According to the Ethiopian Demographic Health Survey (EDHS), a rate of 29 neonatal deaths per 1,000 live births was documented in 2016 which later increased to 30 deaths per 1,000 live birth in the 2019 report. Moreover, 42% of under-five mortalities in Ethiopia are attributable to neonatal deaths (9–11).

Although developing countries have been prioritized to achieve the global target of reducing neonatal death; after comprising 25% of total births and 50% of total deaths in 2013, it is projected that sub-Saharan Africa will contribute 33% of total births and 60% of total deaths by 2030 (12, 13). On the current trend, it has been estimated that more than 60 countries will miss the Sustainable Development Goal (SDG) target of reducing NM to at least as low as 12 deaths per 1,000 live births by 2030. Surprisingly, approximately half of these countries, which carry approximately 80% of the burden, will not reach the target even by 2050 (14).

To alleviate this issue and achieve the goal, in line with WHO global strategy, Ethiopia has endorsed The National Newborn and Child Survival Strategy, which is part of the Health Sector Transformation Plan I (HSTP-I), as well as a targeted reduction of neonatal mortality in HSTP-II (15, 16). However, despite these efforts, NMR continues to be a significant problem in Ethiopia. A better understanding of the timing and proximate determinants of neonatal mortality is, therefore, crucial in designing timing-relevant intervention strategies that will reduce the burden of neonatal mortality. Thus, the current study aimed to assess the time to death of neonates and identify predictors of neonatal mortality.

## Methods and materials

### Study design and setting

An institution-based prospective cohort study design was employed from 1 May to 30 July 2022 in the Silt’e zone, which is 1 of the 14 zones in the Central Ethiopia region and located approximately 172 km away from Addis Ababa (the capital city of Ethiopia). The study was conducted in two phases: the institution phase and the follow-up community phase. The institution phase was conducted in four hospitals (Werabe Comprehensive, Alemgebeya, Kibet, and Tora Hospitals) and two health centers under each hospital. The community follow-up phase was then conducted for all the neonates born in the attended deliveries at these institutions.

### Population

**Source population:** All term deliveries in the Silt’e zone.

**Study population:** The study populations were the sampled term pregnant women and their respective neonates who were available during the data collection period.

**Inclusion criteria:** Term pregnant mothers who had resided in the catchment area for more than 6 months with their neonates and who gave live birth in the study area between 1 May and 30 July 2022 were included in the study.

**Exclusion criteria:** Mothers who were unable to hear or speak and mothers with psychiatric illness were excluded from the study.

### Sample size and sampling technique

The sample size was calculated using the double population proportion formula in STATA version 14.1 with an event proportion of 41 deaths per 1,000 live births, a hazard ratio (HR) of 2.8 for postnatal care, 2.4% withdrawal, and a design effect of 1.5. The final sample size was 1,112 (17). All the hospitals in the zone were included and two health centers under each hospital were selected using a simple random sampling method. Consecutive samples of term deliveries were taken according to their order of delivery. The community follow-up phase was then conducted on the neonates born in the attended deliveries at these institutions.

### Data collection tool and follow-up procedures

Data were prospectively collected using pre-tested interviewer-administered structured questionnaires adapted from different sources. The indicators for the wealth index were adapted from the EDHS (18). Indicators for neonatal care practices were adapted from the WHO minimum neonatal care packages (19). Moreover, the questionnaire contains pregnancy, antenatal care (ANC), and delivery-related questions. All the questionnaires were prepared in English, translated into Amharic, and used to collect the data. Multi-lingual midwives who could speak Siltigna and Amharic and had previous experience in similar areas and supervisors participated in the data collection. Data were collected in three phases. Data collectors obtained informed consent and performed interviews to collect data on the socio-demographic variables, pregnancy, ANC, delivery, and birth outcome during the initial visit. Neonatal morbidity, postnatal care, and other relevant data including survival status were then obtained in a home visit in weeks 1 and 4.

### Study variables

#### Outcome variable

**Survival status**: The event of interest was neonatal death, i.e., the death of a neonate within 28 days of life according to the report of the mother who participated in the study. Other events for this variable included “censored” or “survived” (17).

#### Predictor variables

**Socio-demographic and economic factors**: Place of residence, marital status, education status of mother, wealth index, and occupational status of the mother and father.

**Maternal factors**: Age at childbirth, gravidity, maternal complication during pregnancy delivery and immediately post-partum, ANC visit, place and type of delivery, and postnatal care.

**Neonatal factors**: Birth size; birth order; initiation of exclusive breastfeeding (EBF); neonatal complications such as tachypnea, fever, and hypothermia; and APGAR score.

### Measurement

**Neonatal death:** The death of the neonate was recorded according to the maternal report. The mother was asked about the status of her baby during an interview and death was declared when the mother reported the baby was dead.

**Birth weight:** The birth weight of the newborn was measured immediately after delivery within 15 min. It was recorded by weighing the newborn in light clothes using digital scales that read with 10 g precision. To take a measurement, the scales were placed on a level surface, cleared of any objects, and set to zero before each measurement.

### Operational definition

**Neonatal mortality**: Death of a newborn within 28 days of their birth categorized as “yes” if dead and “no” if censored (5).

**Early neonatal mortality**: Dying before 7 completed days of life (5).

**Late neonatal mortality**: Dying between 7 completed days and before 28 completed days (5).

**Term pregnancy**: A pregnancy between 37 and 42 completed weeks of gestation (17).

**Birth weight**: Categorized into **“**low birth weight” for a weight less than 2,500 g; “normal birth weight” for the weight category of 2,500–4,000 g; or “high birth weight” for a weight greater than 4,000 g (17).

**Maternal complication**: Mother experiencing one or more complications such as prolonged labor and/or excessive bleeding before, during, or after delivery, and/or diagnosed with hypertensive disorders of pregnancy (HDP), i.e., pre-eclampsia/eclampsia, and/or a premature rupture of membranes (PROM) (20).

**APGAR score**: Measured in the 1st minute after birth and classified into low (1–3), intermediate (4–6), or high (7–10, 21).

### Data quality assurance

Data quality assurance was conducted through the careful translation and back translation of the data collection tool. After its consistency was assured, a 2-day training course on the instrument and about data collection and supervision was provided for the data collectors and supervisors by the principal investigator. In addition, birth weight measurement quality was assured by using electronic scales and training with supportive supervision as per the recommendations of previous studies on birth weight quality assurance mechanisms (22). The training on how to take birth weight provided to the data collectors involved practice weighing 10 babies with the principal investigator following the training session. Both intra-observer and inter-observer technical error of measurement (TEM) were computed. The intra-observer TEM ranged from 0.09 to 0.1, and the inter-observer TEM ranged from 0.07 to 0.1 every morning, following an instrument relocation, calibration and validation were performed using a standard 1 kg weight. The calibration result was recorded on a sheet containing a table for the report. The average reading was 1 ± 8 kg. A week before actual data collection, pretesting of the tool was conducted in 10% of the sample size in the woredas selected for this study. Data were checked daily before entry for completeness during the actual data collection period and then double data entry was used to check consistency.

### Data processing and analysis

Data were entered into EpiData version 3.1 and then transferred to STATA version 14 to be coded, cleaned, and analyzed. Variables were described using mean with standard deviation (SD) or median with interquartile range (IQR) for continuous data, and frequency with proportion for categorical data, accordingly. The Kaplan–Meier survival curve was used to show the pattern of neonatal death in 28 days. The neonatal outcome variable was examined against all the predictor variables using bi-variable analysis and those with a *p-*value ≤0.25 were considered to be a candidate for multi-variable analysis. The Cox proportional hazard regression model was fitted to see the independent and adjusted relationships of different predictors with neonate survival. Mortality risk was presented with a hazard ratio and 95% confidence interval (CI). A *p*-value <0.05 was considered statistically significant. Multicollinearity was considered with a variance inflation factor (VIF) greater than 10 before interpreting the final output. Model fitness was checked by plotting a curve of –ln[−ln(survival probability)] against ln(survival time) as well as by the global goodness of fit test.

### Ethics approval

Ethical clearance (Ref. No. ወራ/ዩ/ም/ህ/ዳ/1/57/14) was obtained from the Werabe University Ethical Review Board (ERB). It was subsequently submitted to the Silt’e Zone Health Office to get permission. Following this, the selected participants were contacted by an assigned person; consent to collect anthropometric, vital signs, and other data of the newborns, and a written informed consent for maternal-related data from each participant/mother/caregiver were obtained after detailed information was given regarding the research objectives, data collection methods, and the right to give, deny, and withdraw from the interview and/or from the study at any time. All methods were performed in accordance with the relevant guidelines and regulations.

## Results

### Participants characteristics

**Figure 1:**
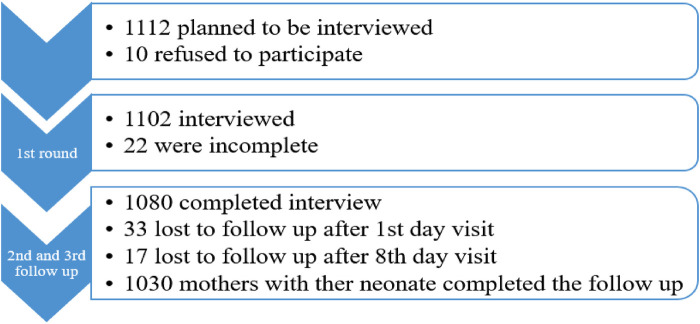
Data collection result for survival status and predictors of mortality among neonates in Silt’e zone, Central Ethiopia, in 2022.

In total, 1,080 term neonates were followed for a total of 27,643.6 neonatal days. In this study, 1,112 term deliveries were planned to be included and followed up to 28 days. Initially, 10 refused to participate and 1,102 were included in the study. Later, 22 questionnaires were incomplete and discarded from the analysis (Figure 1).

#### Socio-demographic characteristics

The mean ± SD maternal age of the respondents at the current birth was 26.8 ± 4.9 years with the majority of them (91.4%) between 20 and 35 years old. Approximately 39% of them had received a primary school level of education. Moreover, 6.4% of the mothers were employed in work that could generate an income. Approximately half of the fathers were farmers, and approximately 26% fell in the fourth quartile wealth category (Table 1).

**Table 1:** Socio-demographic characteristics of the participating mothers in Silt’e zone, Central Ethiopia, in 2022.

Variable	Category	Frequency	Percentage
Maternal age at current birth	<20	49	4.5
	20–35	987	91.4
	>35	44	4.1
Marital status	Single	49	4.5
	Married and/or live with a partner	1,008	93.3
	Widowed/divorced	23	2.2
Maternal educational level	No formal education	468	33.3
	Primary education	419	38.8
	Secondary and preparatory education	129	11.9
	Higher education	64	5.9
Maternal occupation	Housewife	821	76.0
	Farmer	138	12.8
	Employed	69	6.4
	Self-employee	52	4.8
Father occupation	Farmer	534	49.4
	Employed	261	24.2
	Self-employee	285	26.4
Wealth index	First quartile	276	25.6
	Second quartile	263	24.4
	Third quartile	256	23.7
	Fourth quartile	285	26.4

#### Pregnancy-related characteristics

Among the total respondents, 929 (86%) were multigravida. The previous pregnancy outcomes of the multigravida women included 127 (13.7%) previous abortions while 61 (6.6%) had a previous neonatal loss and 84 (9.1%) had experienced a stillbirth. In this study, 28 (2.6%), 68 (6.3%), and 4 (0.4%) mothers were ever smokers, chewers, and alcohol drinkers of any amount in their lifetime, respectively. Among the mothers who attended the ANC clinic, 225 (27.9%) of them had ≥4 ANC visits and 336 (41.8%) received their ANC in the hospital. Finally, 712 (88.4%) mothers had received at least one dose of tetanus toxoid (TT) in their current pregnancy while 90% had one or more doses of TT vaccination in their lifetime (Table 2).

**Table 2:** Pregnancy-related characteristics of the participating mothers in Silt’e zone, Central Ethiopia, in 2022.

Variable	Category	Frequency	Percentage
Attend ANC clinic	Yes	804	74.4
	No	276	25.6
Place of ANC care	Hospital	336	41.8
	Health center	369	45.9
	Health post	98	12.2
Number of ANC visits	Less than four	579	72.0
	Four or more	225	28.0
Counseling services they received	Hygiene and self-care	461	57.3
	Breast feeding	491	61.1
	Child nutrition	508	63.2
	Infant immunization	528	65.7
Received iron tablet	Yes	663	82.5
	No	141	17.5
Received TT vaccine in the current pregnancy	Yes	712	88.4
	No	92	11.6
Doses of TT in current pregnancy	One	352	49.4
	Two or more	360	50.6
Ever received a TT vaccine	Yes	982	90.9
	No	98	9.1
Doses of TT ever received	One	659	67.1
	Two or more	323	32.9
MUAC (cm)	<23	141	13.1
	≥23	939	86.9

MUAC, mid upper arm circumference.

#### Delivery-related characteristics

Of the total participants, 149 (13.8%) deliveries had one or more delivery complications (22.1% HDP, 34.2% were multiple-gestation births, and 38.9% were PROM). Approximately 627 (58.1%) newborns were delivered at the hospital; furthermore, 998 (92.4%) were vaginal deliveries (Table 3).

**Table 3:** Delivery-related characteristics of the participating mothers in Silt’e zone, Central Ethiopia, in 2022.

Variable	Category	Frequency	Percentage
Place of delivery	Hospital	627	58.1
	Health center	453	41.9
Reason for visit	Self-initiated	1,020	94.4
	Referral from other health facility	60	5.6
Social accompany	Accompanied by others	1,063	98.4
	Not accompanied by others	17	1.6
Diagnosed delivery complication	Yes	149	13.8
	No	931	86.2
Type of complication	HDP	33	22.1
	Prolonged labor	29	19.5
	PROM	58	38.9
	Multiple gestations	51	34.2
	Others	21	14.1
Type of delivery	Vaginal	998	92.4
	Cesarean section	82	7.6
Birth order in multiple gestations	First	34	66.7
	Second or more	17	33.3

#### Newborn characteristics

The male-to-female birth ratio was 1:1.2. The mean birth weight was 3,270 ± 494.7 g with 91.9% of the weights falling between 2,500 and 4,000 g and the mean temperature was 36.8 ± 0.3°C. Newborns with a 1st minute APGAR score of 7–10 comprised 96.1%, 1.4% scored 4–6, and the rest comprised 0%–3%. In total, 26 (2.4%) newborns had a congenital deformity, 990 (91.7%) started breastfeeding within 1 h and 1,024 (94.8%) received colostrum on the 1st day. Of the total deliveries, only 460 (42.6%), and 512 (47.4%) newborns received BCG and OPV0 vaccines immediately after birth, respectively. Moreover, only 435 (40.3%) of them received cord ointment (Table 4).

**Table 4:** Newborn characteristics of participating neonates in Silt’e zone, Central Ethiopia, in 2022.

Variable	Category	Frequency	Percentage
Sex	Female	589	54.5
	Male	491	45.5
Birth weight (g)	<2,500	50	4.6
	2,500–4,000	992	91.9
	>4,000	38	3.5
Temperature (°C)	<36.5	55	5.1
	≥36.5	1,025	94.9
1st minute APGAR score	0–3	27	2.5
	4–6	15	1.4
	7–10	1,038	96.1
Congenital deformity	Yes	26	2.4
	No	1,054	97.6
Resuscitated	Yes	172	15.9
	No	908	84.1
Received TTC	Yes	963	89.2
	No	117	10.8
Received Vitamin K injection	Yes	873	80.8
	No	207	19.2
Received umbilical ointment	Yes	435	40.3
	No	645	59.7
Received OPV0	Yes	512	47.4
	No	568	52.6
Received BCG	Yes	460	42.6
	No	620	57.4
Breastfeeding initiated within 1 h	Yes	990	91.7
	No	90	8.3
Baby received colostrum	Yes	1,024	94.8
	No	56	5.2

TTC, tetracycline.

#### Survival status and outcome of the follow-up

**Figure 2:**
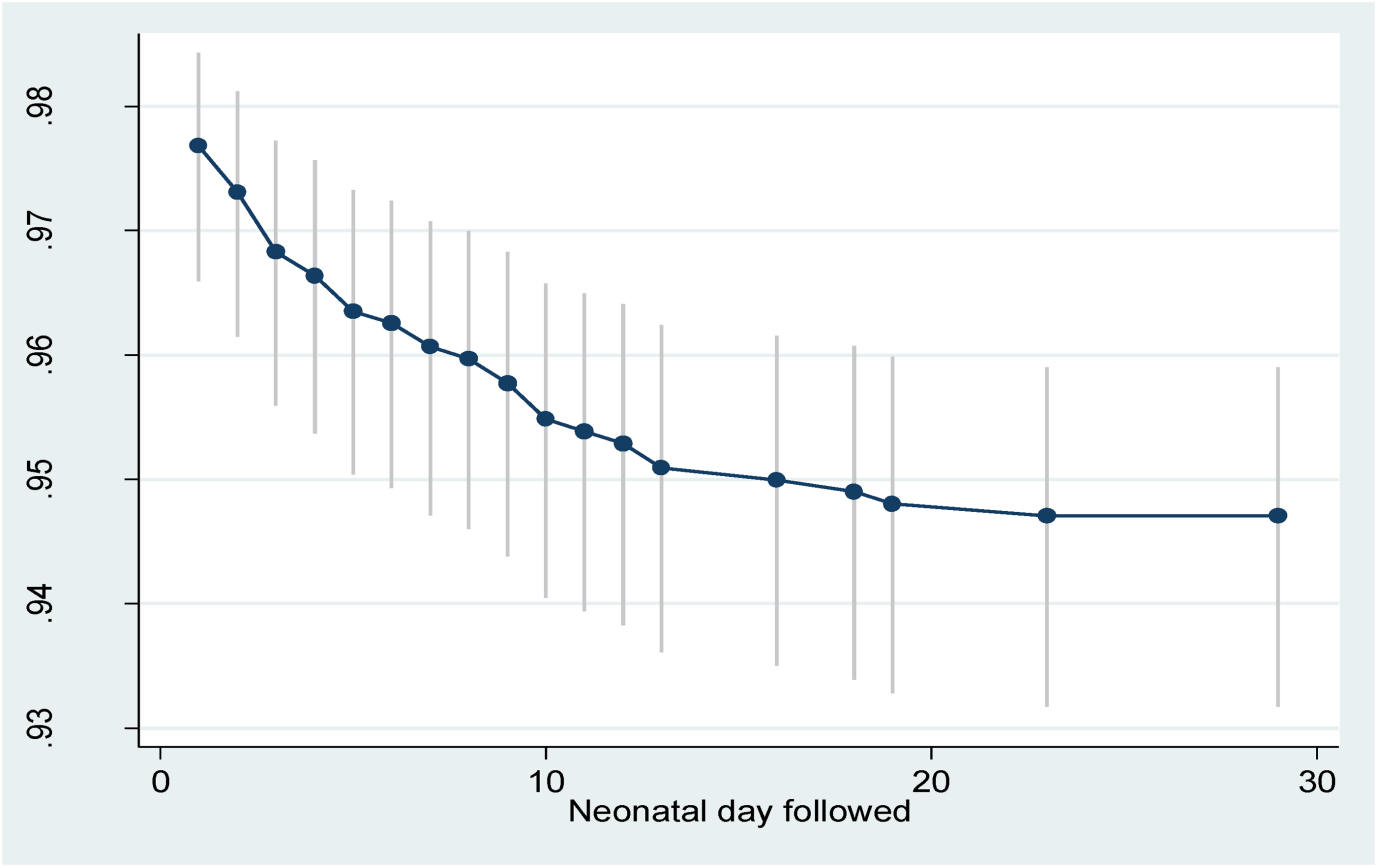
Survival probability with 95% confidence interval of neonates in Silt’e zone, Central Ethiopia, in 2022.

The total follow-up days was 27,643.6 neonatal days. Of those who completed the study, 56 neonates died (a cumulative incidence of 52 deaths/1,000 live births) which provided a cumulative probability of surviving 28 days among the neonates of 95% (Figure 2). The overall incidence rate was 2.02 deaths per 1,000 neonatal days. Of the deaths, 25 (44.6%) occurred in the first day, 42 (75.0%) deaths occurred in the first week, and 14 (25.0%) died between 8 and 28 days. Furthermore, the cumulative hazard during the early neonatal period (days 0–7) was 0.039 while in the late neonatal period (days 8–28) it was 0.014.

### Predictors of neonatal mortality

#### Bi-variable analysis

In the bi-variable association analysis between survival status and socio-demographic characteristics, all variables except marital status and wealth index, were insignificantly associated at a *p*-value of ≤0.25. Regarding pregnancy and ANC-related characteristics, history and the number of TT vaccines ever injected, previous history of neonatal death, gravidity, presence of complication in current pregnancy or delivery, and history of ever smoking and alcohol drinking showed crude associations with neonatal death. In addition, referral history, delivery mode, and birth order of a baby in multiple gestations were shown to have an association. Moreover, sex, 1st minute APGAR score, resuscitation after birth, BCG vaccination immediately after birth, initiation of breastfeeding within 1 h, and receiving colostrum were newborn characteristics that had a crude association at a *p*-value of ≤0.25 (Table 5).

**Table 5:** Predictors of mortality among neonates in Silt’e zone, Central Ethiopia, in 2022.

Variable	CHR (95% CI)	*p*-value	AHR (95% CI)	*p*-value
Marital status				
Single/lives with no partner^a^	1		1	
Married or live with partner	0.17 (0.04–1.82)	0.172	0.37 (0.05–2.92)	0.346
Wealth index				
Fourth quartile	1		1	
First quartile	2.61 (1.15–5.94)	0.021	1.61 (0.67–3.85)	0.285
Second quartile	1.20 (0.46–3.11)	0.708	0.87 (0.32–2.33)	0.779
Third quartile	2.70 (1.18–6.18)	0.018	1.78 (0.74–4.27)	0.196
TT vaccination history				
No	1		1	
Yes	0.52 (0.25–1.06)	0.071	0.88 (0.39–2.03)	0.773
Number of TT vaccine received				
Two or more	1		1	
Less than two	2.12 (1.04–4.44)	0.039	2.05 (0.90–4.63)	0.086
History of smoking cigarette ever				
No	1		1	
Yes	2.28 (0.71–7.31)	0.164	2.19 (0.57–8.48)	0.256
History of drinking alcohol ever				
No	1		1	
Yes	1.81 (0.78–4.22)	0.171	2.00 (0.77–5.22)	0.156
Reason for visit				
Referral from other health facilities	1		1	
Self-initiated	0.40 (0.18–0.89	0.024	0.58 (0.23–1.47)	0.255
Gravidity				
Multigravida	1		1	
Primigravida	1.55 (0.80–3.00)	0.191	1.37 (0.64–2.92)	0.419
History of neonatal death				
No	1		1	
Yes	6.84 (3.78–12.35)	<0.001	4.03 (2.28–9.52)	<0.001**
Pregnancy complication				
No	1		1	
Yes	1.97 (0.84–4.59)	0.117	1.06 (0.41–2.74)	0.909
Diagnosed complication during delivery				
No	1		1	
Yes	7.48 (4.42–12.63)	<0.001	3.08 (1.12–8.25)	0.029*
Delivery mode				
Vaginal	1		1	
Caesarian section	7.17 (4.12–12.48)	<0.001	1.66 (0.64–4.33)	0.297
Birth order^b^				
Second or more	1		1	
First	0.39 (0.13–1.16)	0.092	0.37 (0.10–1.31)	0.123
Sex of the newborn				
Male	1		1	
Female	0.39 (0.22–0.68)	0.001	0.45 (0.25–0.84)	0.011*
Weight of the newborn (g)				
<2,500	1		1	
2,500–4,000	0.28 (0.13–0.58)	0.001	0.27 (0.11–0.63)	0.003**
>4,000	0.15 (0.02–1.21)	0.074	0.15 (0.02–1.37)	0.092
1st minute APGAR score				
7–10	1		1	
0–3	11.1 (5.56–22.18)	<0.001	3.11 (1.23–7.82)	0.016*
4–6	9.84 (3.89–29.92)	<0.001	5.34 (1.63–17.51)	0.006*
Resuscitation received				
No	1		1	
Yes	1.95 (1.08–3.51)	0.027	1.60 (0.78–3.26)	0.196
Received BCG immediately				
No	1		1	
Yes	0.63 (0.36–1.10)	0.102	0.89 (0.46–1.72)	0.732
Started breast feeding within 1 h				
No	1		1	
Yes	0.63 (0.28–1.39)	0.249	0.87 (0.21–3.67)	0.851
Received colostrum immediately				
No	1		1	
Yes	0.56 (0.22–1.39)	0.209	1.10 (0.28–5.31)	0.825

CHR, crude hazard ratio.

^a^ Single is a combination of unmarried, divorced, and widowed mothers.

^b^ Birth order is the order of delivery in multiple-gestation deliveries.

* *p*-value < 0.05; **p-value < 0.005.

#### Multi-variable analysis

Predictors of neonatal mortality were assessed by fitting a Cox proportional hazard model using the variables that showed a crude association. None of the socio-demographic characteristics were significantly associated with neonatal death. History of and doses of TT vaccine in the mother’s lifetime was also shown to have a non-significant association in the multi-variable analysis.

Regarding pregnancy-related characteristics, neonates born of mothers with a history of previous neonatal death were four times more likely to die than their counterparts [adjusted hazard ratio (AHR) = 4.03; 95% CI = 2.28–9.52; *p* < 0.001]. In addition, the neonates of mothers who had delivery-related complications were approximately three times more likely to experience neonatal death than neonates delivered without delivery complications (AHR = 3.08; 95% CI = 1.12–8.25; *p* = 0.029).

**Figure 3:**
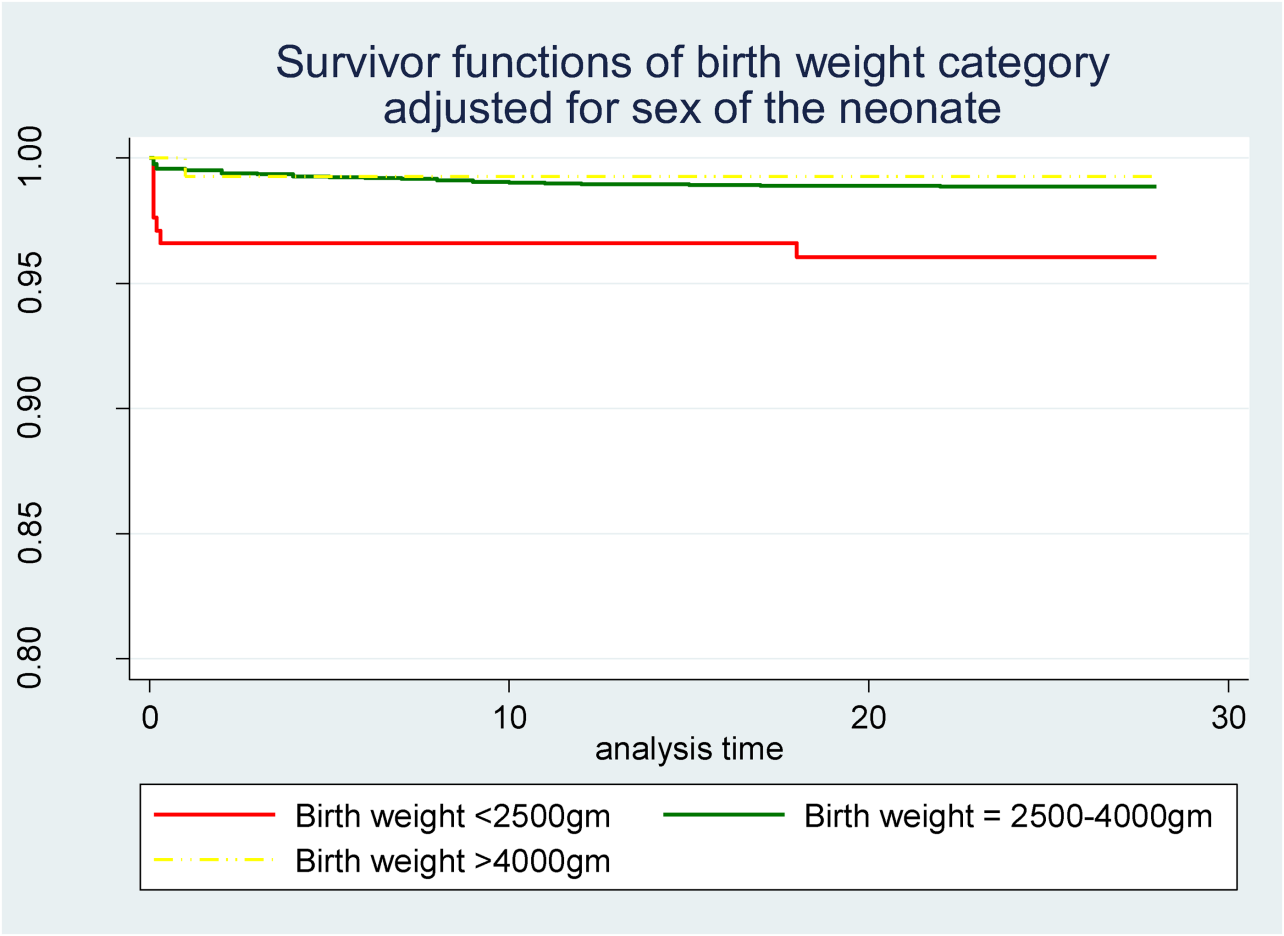
Survivor function of neonates born with different weights in Silt’e zone, Central Ethiopia, in 2022.

Newborn characteristics were also found to be significant predictors of neonatal mortality. In this study, females were found to be at 54% less risk than males (AHR = 0.46; 95% CI = 0.25–0.84; *p* = 0.011). Newborn weight was also another significant predictor in which newborns in the weight category of 2,500–4,000 g were 73% less likely to die in the neonatal period than low-birth-weight (<2,500 g) newborns (AHR = 0.27; 95% CI = 0.11–0.63; *p* = 0.003). However, the effect of high birth weight (>4,000 g) was found to be statistically non-significant compared to low birth weight. A survivor function based on delivery weight also showed a lower survival probability among neonates with low birth weight (<2,500 g) than those with a normal birth weight (2,500–4,000 g) as well as those with high birth weight (>4,000 g) throughout the neonatal period (Figure 3).

Although both the APGAR score and newborn resuscitation showed a significant bi-variable association with neonatal survival status, in an adjusted analysis, only the 1st minute APGAR score category was a significant predictor of neonatal mortality. Neonates whose APGAR score was between 0 and 3 or 4 and 6 were found to be three times (AHR = 3.11; 95% CI = 1.23–7.82; *p* = 0.016) or five times (AHR = 5.34; 95% CI = 1.63–17.51) more at risk of experiencing neonatal death respectively than newborns whose APGAR score was 7–10, respectively (Table 5).

#### Model fitness

**Figure 4:**
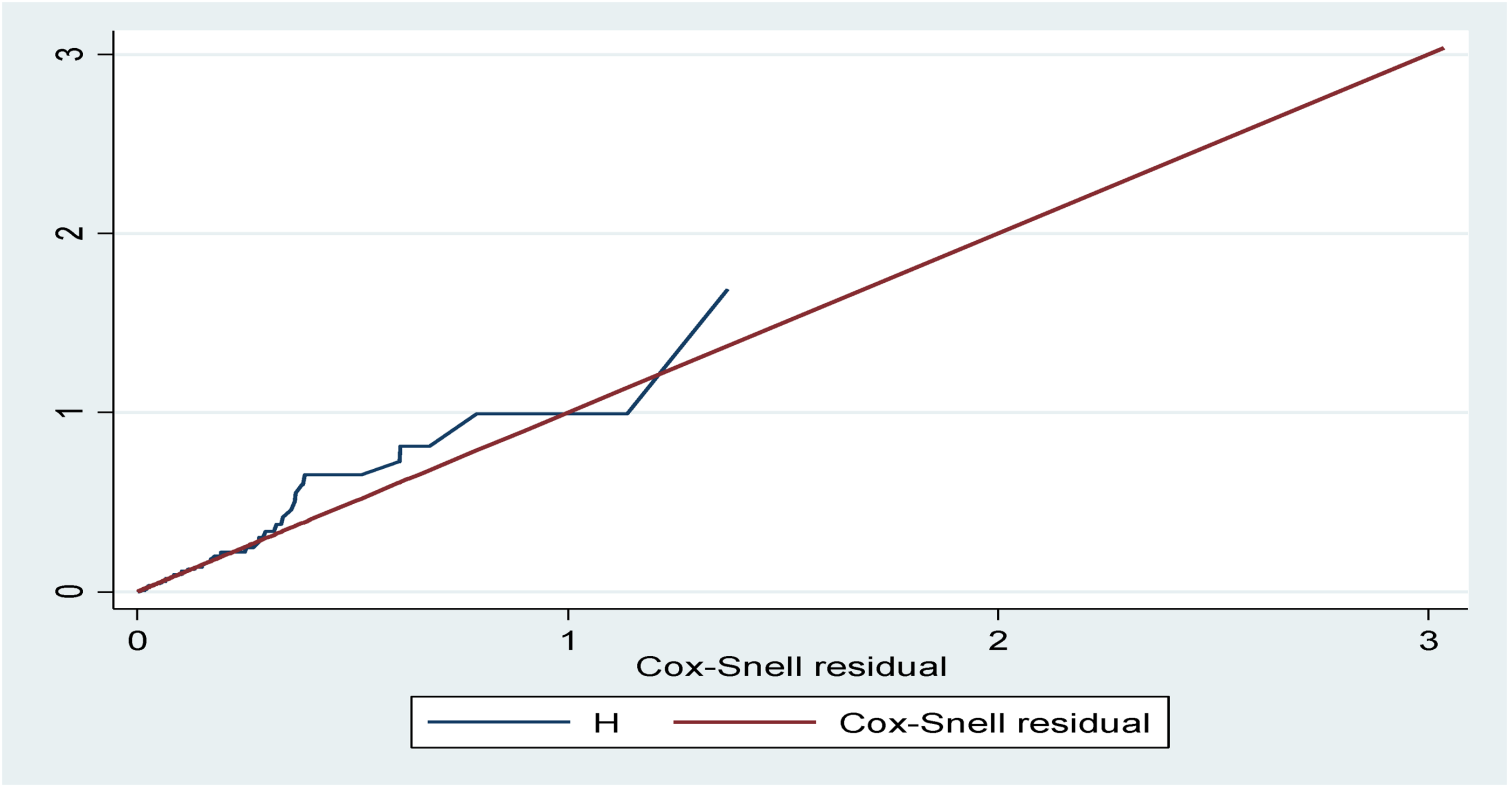
Cox–Snell residual plot of neonatal survival data.

The model fitness was checked for the bi-variable and multi-variable analyses by plotting a curve of –ln[−ln(survival probability)] against ln(survival time) using the global Schoenfeld residual test and Cox–Snell residuals plot. The Schoenfeld residual test showed a *p*-value of 0.739, which was non-significant, and the Cox–Snell residuals graph also followed the 45° hazard curve which showed that the model was a good fit (Figure 4).

## Discussion

The study assessed survival status and predictors of mortality among neonates. Birth weight, sex of the newborn, and APGAR scores predicted neonatal survival. In addition, a poor outcome in a previous pregnancy and a current complicated pregnancy were also found to increase the risk of neonatal death.

Based on the result, the Silt’e zone has a neonatal mortality rate of 52 deaths/1,000 live births. Compared to the global rate in 2019, the result from this study was higher (23). This figure was also higher than the results from studies conducted in Kenya and South Africa (24). Moreover, this result was higher than that in EDHS 2019 (11), a study conducted in Jimma zone (20) and the Aroressa district (17). The possible reasons might be study area and design differences as well as healthcare delivery characteristics. However, this result was lower than a recent study conducted in the Tigray region (5). Although the explanation for the difference in mortality might be as mentioned above, the finding indicates our study area needs attention concerning neonatal death.

In this study, close to half and three-quarters of the deaths occurred on the first day and in the first week, respectively. This finding was similar to previously conducted studies in Jimma, Tigray, and Aroressa (5, 17, 20). The reason might be that in developing countries, the majority of neonatal mortalities are related to immediate prepartum, intrapartum, and immediate newborn care practices. This clearly shows that neonatal survival interventions have to target the intrapartum and the immediate and early neonatal periods. Furthermore, poor-quality antenatal care and a delay in the identification and poor management of complications during pregnancy and birth by health workers might be possible reasons as such factors are common in low- and middle-income countries and have been found to cause neonatal death in previous studies (5).

In this study, a history of previous neonatal death was found to significantly predict neonatal survival. This finding was consistent with previous studies (25–27). The need for special follow-up care and screening during the initial visit for pregnant mothers with a previous history of neonatal death could therefore be another implication of this study. As the WHO ANC high-risk pregnancy definition does not consider previous history of neonatal death as high risk, this study paves the way for including it in the high-risk pregnancy category.

Complicated deliveries (i.e., common maternal complications during labor) were also found to predict the risk of neonatal death in this study. Similar findings were reported in previous studies (5, 20, 28). The reasons could be the low availability and quality of services, including emergency obstetric services, supplemented by poor understanding of mothers with complications and delayed referrals. This is supported by the findings of some studies, which indicated that high NM often occurs because of delivery and pregnancy-related complications and this denotes the quality of healthcare in an area (29). Moreover, our study showed a higher proportion of newborns with lower APGAR scores, which has a clear association with asphyxia and death in complicated deliveries compared to non-complicated deliveries.

Certain newborn characteristics were also found to predict neonatal death. In this study, female neonates were found to have less risk of death than male neonates. This finding was similar to results from South Asia, the Aroressa district, and other national studies (10, 17, 30). One of the explanations for this could be that males are at higher risk of intrauterine growth restriction. In addition, they are at higher risk of respiratory morbidities and gastrointestinal infections, likely due to high testosterone levels that suppress the immune system (30). Although not a direct implication, our study found a slightly higher risk related to a low APGAR score in male compared to female newborns.

In this study, low-birth-weight (<2,500 g) neonates were found to have a higher risk of death than normal-weight newborns. This finding is similar to studies conducted in Tigray, Jimma, Aroressa, and Felege Hiwot (17, 20, 28, 31). This could be due to that low-birth-weight newborns are at higher risk of infection (32).

Low and intermediate 1 min APGAR scores of both 0–3 and 4–6 were significant predictors of neonatal death and increased the risk of neonatal mortality up to three and fivefold, respectively. This finding was consistent with studies conducted in China, Sweden, and Arba Minch (21, 33, 34). The reason might be that a lower APGAR score, even within the normal range such as 7–9, is strongly associated with an increased risk of neonatal morbidity. Furthermore, other studies have found progressively higher relative odds of infections, asphyxia-related complications, neonatal hypoglycemia, and respiratory distress to be associated with lower APGAR scores (33).

### Strength and weakness

As most of the studies conducted in the area were retrospective and cross-sectional study designs, the current study is a good source of data for time to neonatal death. This study was a prospective cohort study which is appropriate for time-to-death analysis as it minimized biases due to memory loss and other characteristics of retrospective and cross-sectional studies. As a drawback, although data collectors tried to minimize it, maternal pregnancy history and variables in the past were assessed using self-report which might contain a memory-related information bias. In addition, there were some time-variant characteristics that are believed to predict the survival of neonates, such as infection/morbidity and others, which need to be investigated using other analysis methods that are not covered in the current study.

## Conclusion and recommendation

This study assessed neonatal survival status, time to death, and the risk birth weight posed for neonatal survival. Our finding showed that neonatal mortality was higher than most of the other study areas, and thus needs strict attention, programs, and interventions based on the identified predictors. Low-birth-weight babies are found to struggle to survive the neonatal period. Male sex and a lower APGAR score at 1 min were also newborn characteristics that increased the risk of neonatal mortality. Newborns of mothers with a previous history of neonatal death and complicated delivery were at higher risk of neonatal death. Therefore, health offices, health professionals, non-governmental organizations, and other stakeholders should work in harmony to target both pre- and postnatal period factors to reduce neonatal mortality in the Silt’e zone. Furthermore, promoting maternal nutrition to ensure normal birth weight for the newborn would thereby improve neonatal survival.

## Data Availability

The original contributions presented in the study are included in the article/Supplementary Material, further inquiries can be directed to the corresponding author.
